# 816 Up-regulation of Immune Related Key Genes in Burn Induced Cardiac Dysfunction

**DOI:** 10.1093/jbcr/irac012.364

**Published:** 2022-03-23

**Authors:** Keyan Mobli, Jake J Wen, Geetha Radhakrishnan, Ravi S Radhakrishnan

**Affiliations:** UTMB, Galveston, Texas; University of Texas Medical Branch, Galveston, Texas; UTMB, Galveston, Texas; University of Texas Medical Branch, Galveston, Texas

## Abstract

**Introduction:**

The cytokine cascade is a prime factor in inflammation following burn injury, leading to cardiac dysfunction. Studies on immune-related key cardiac genes after burn injury remain limited. The TLRs act individually or as heterodimers, interacting with adaptor proteins to initiate MyD88 or TICAM1 (TRIF) dependent responses. This results in signaling cascades through NFkB, which activates downstream JNK/p38 signaling or cytokine secretion. Dysregulation of TLR and related signaling pathways has severe consequences and is implicated in autoimmune diseases and chronic inflammation. The objective of this study is to identify the immune-related key genes and explore the pathways by microarray specific to members of the TLR signaling family as well as adaptor and effector proteins, and downstream members of TLR activation including the NFkB, JNK/p38, IRF and JAK/STAT signaling pathways.

**Methods:**

The microarray was applied to profile expression of 84 genes inTLR-mediated signal transduction and innate immunity. The Qiagen web-based tools in GeneGlobe Data Analysis Center was used to analyze burn-induced up-regulation of these genes. String-DB version 11.5 was employed to analyze differentially expressed gene function and interaction. Real-time qPCR was utilized to validate the data. GO enrichment analysis (FDR< 0.05) using "clusterProfiler" predicted the roles of immune-key genes in the 3 aspects of biological processes (BP), cellular components (CC), and molecular functions (MF). The biological pathway analysis was performed to obtain the pathways. The protein-protein interaction (PPI) network was built using "Multiple Protein" of the string database (http://stringdb.org/, version 15.0). The DEGs were visualized by using the qPCR.

**Results:**

32 up-regulated genes were identified. Functional enrichment analysis showed that these genes were enriched in cytokine receptor activity, immune receptor activity, and integrin family cell surface interactions.14 of 32 genes belong to the TLR signaling pathway; 5 of 32 genes to cytokines and inflammation; 5 of 32 genes belong to apoptosis; 6 in the MARP signaling pathway; 4 genes belong to the IL-1 signaling pathway; 4 genes in the IL-6 signaling pathway); 3 genes in toIL-2 signaling pathway and 3 genes belong to oxidative stress responses. Process study demonstrated the genes are associated with regulation of cytokine production (GO:0001817), immune response (GO:0006955), and defense response (GO:0006952).

**Conclusions:**

This study provides evidence that microarray plays a role in identification of burn-induced cardiac dysfunction DEGs. Deep analysis of up-regulated genes after burn injury supported the idea that the proinflammatory cascade results in cardiac inflammation.

